# Conservative Managing of Bezoar in Giant Hiatus Hernia Causing Gastric Outlet Obstruction During the COVID-19 Pandemic

**DOI:** 10.1007/s11695-021-05250-y

**Published:** 2021-02-12

**Authors:** Hany Khalil, Chetan Parmar, Pratik Sufi

**Affiliations:** The Whittington Hospital, London, UK

**Keywords:** Bezoar, COVID-19, Giant hiatus hernia, Gastric outlet obstruction

## Background

There are four different types of bezoar according to their composition, and the commonest type amongst them all is phytobezoar, which accounts for nearly 40% of the bezoars reported. Other types are trichobezoars, lactobezoars and pharmacobezoars [1]. Phytobezoars consist of indigested food particle, fruit fibers, seeds and pulps.

The vast majority of phytobezoar is detected in stomach (78%), however 17% of this pathology can be found in the small intestine [1]. They have been reported in the stomach after surgery like Nissen Fundoplication [R], Billroth I resections, and in the ileum after Billroth II resections causing intestinal obstruction [2]. Phytobezoar in hiatus hernia (HH) is an uncommon finding [3]. The management of this condition becomes even more challenging if it presents during the peak of COVID-19 pandemic as the mortality is high [4].

We report successful conservative management of a patient with a large phytobezoar in a giant hiatus hernia [5] causing gastric outlet obstruction.

## Case Presentation

A 69-year-old female patient with a known HH, treated hypertension and chronic obstructive pulmonary disease (COPD) presented to the hospital with vomiting and inability to tolerate food or drink. She was dehydrated but her vital observations were stable.

**Fig. 1:**
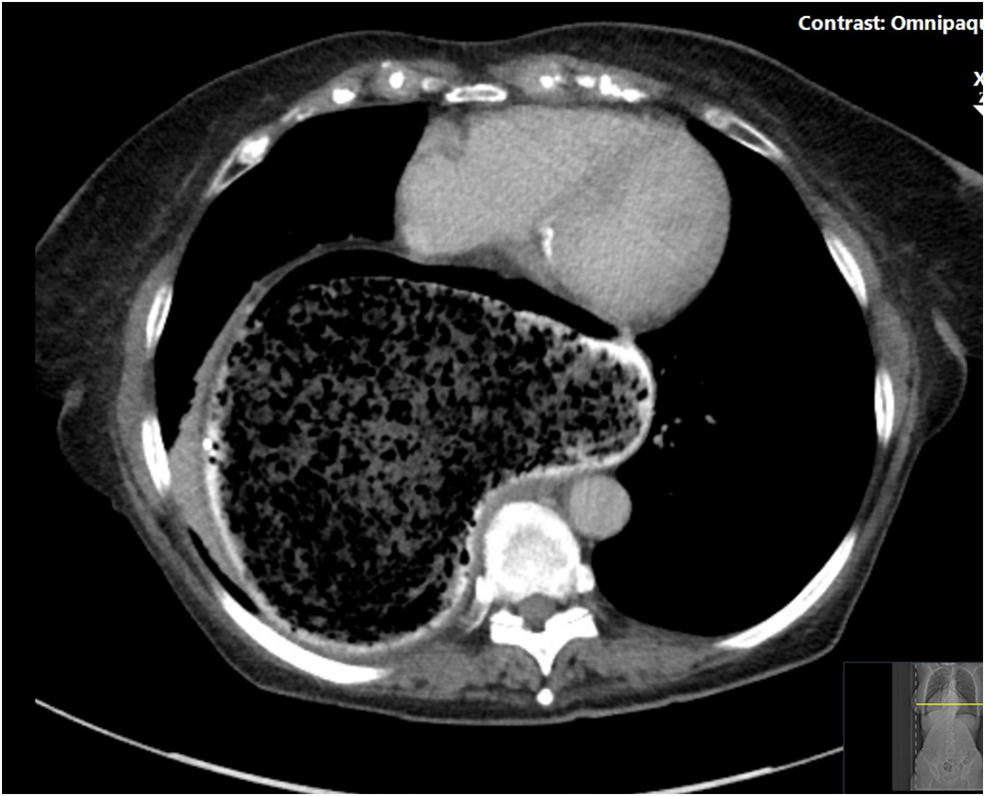
CT scan showing food in the giant hiatus hernia in the chest

**Fig. 2:**
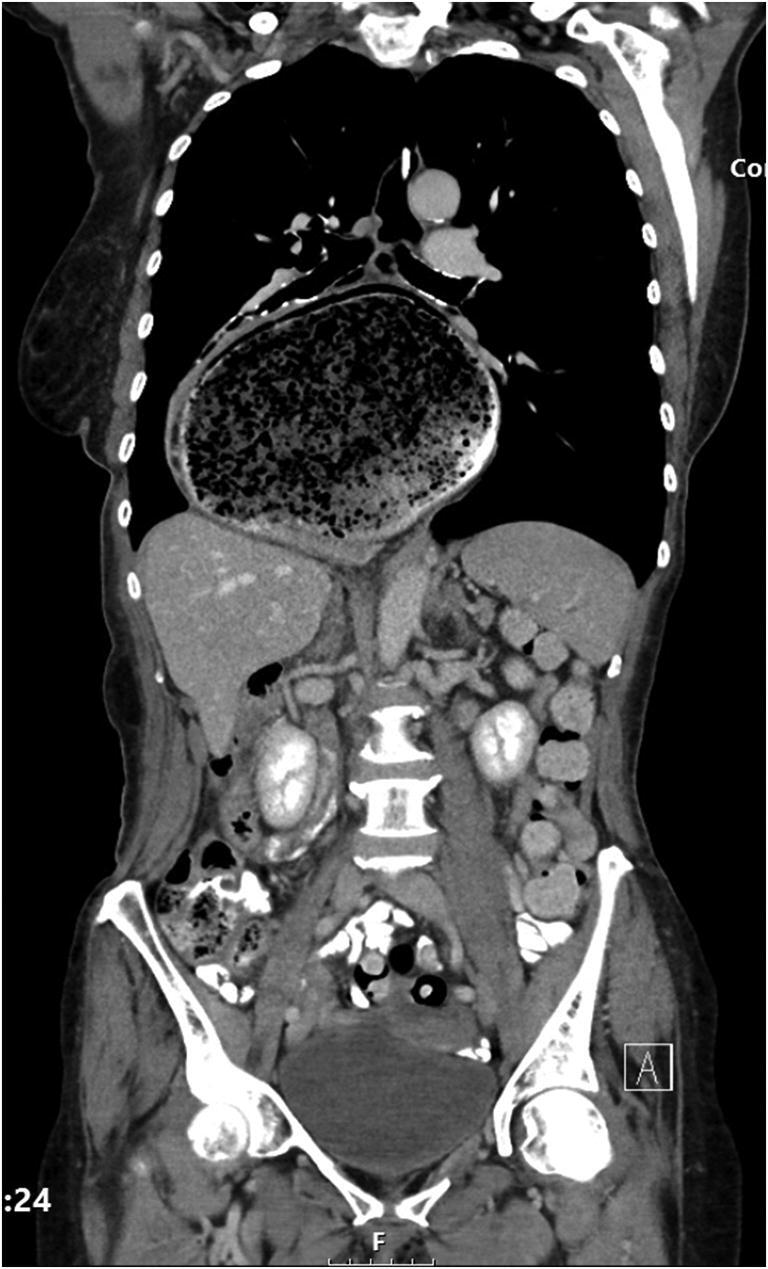
CT scan showing food in the giant hiatus hernia in the chest

**Fig. 3:**
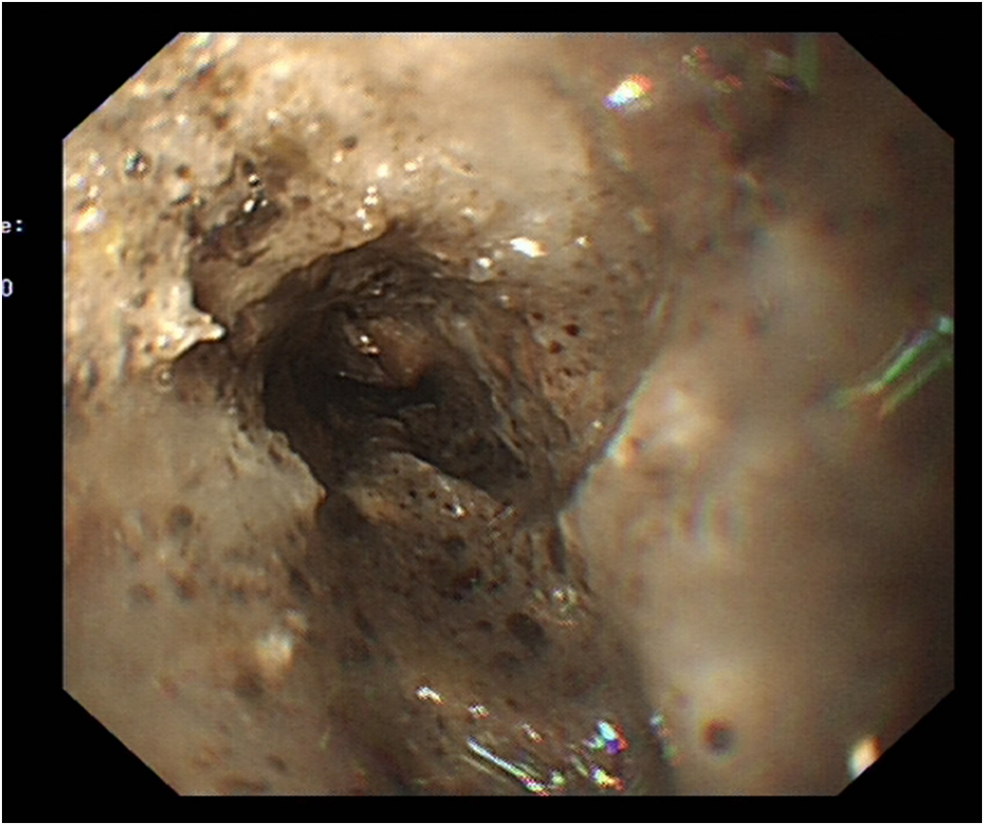
Image of OGD showing phytobezoar in the giant hiatus hernia

A nasogastric tube (NGT) was inserted in the Accident and Emergency (A&E) Department. A computed tomography (CT) scan of the chest, abdomen and pelvis requested. This revealed a giant HH (size; 13 cm × 15 cm × 13 cm, CC × ML × AP respectively) containing a large bezoar, mostly within the right side of the thorax, significantly distended with air and stomach contents and the NGT tracing round the lateral border of the HH (Figs. 1 and 2). The patient presented to the department during the peak of the COVID-19 pandemic in our region. All elective activities were cancelled. Only life-saving and emergency operations and endoscopy were performed as per the national guidelines [4]. The patient was suspected COVID-19 positive (low lymphocyte count and the CT chest showed bilateral, subpleural and peripheral ground-glass opacities) [6]. The patient then underwent a oesophago-gastro-duodenoscopy (OGD) with full personal protective equipment (PPE), on the day after her admission, which revealed a large volume of inspissated food (Fig. 3), causing gastric outlet obstruction, in the hiatus hernia. The procedure was aborted as the patient vomited during the OGD and there was a risk of aspiration, with a plan to repeat the procedure after 48 h with prolonged fasting and optimisation. The patient was advised to drink carbonated drinks, and prescribed erythromycin to facilitate bowel emptying. After 48 h, the patient was reassessed—she clinically felt better and hence it was decided to postpone the OGD. The vomiting subsided within 96 h—the NGT was removed and the patient was served light and soft food, which she tolerated. The patient was seen by a dietitian who advised regarding eating habits in the future. The patient was discharged 5 days after her initial presentation.

### Outcome and follow up

Six months later, the patient was followed up by a telephone clinic, due to the COVID pandemic in the outpatient clinic. The patient will have a repeated OGD as an outpatient investigation to assess the progress of the management.

## Discussion

Giant hiatus hernia generally presents with chest pain, anaemia, dysphagia, weight loss, etc., and phytobezoar has not been reported in these patients previously.

Barium studies, ultrasound and computed tomography (CT) scans are used to diagnose phytobezoar; however endoscopy remains the gold standard investigation because it can direct visualize and treat the condition.

Gastric bezoars can be treated with digestive enzymes or can be broken up by endoscopy; the surgical option is rarely indicated. Intestinal bezoars can be treated by long tube and enzyme instillation, but usually require laparotomy.

The presence of a large bezoar in the hiatus hernia causing obstruction is a rare condition. Conservative management may be successful in treating the patient’s acute symptoms, provided that the patient is vitally stable, with Emergency surgery reserved for those who fail. Another option for management of phytobezoar is the endoscopic removal which was first performed by McKechnie in 1972 [7, 8]. Fragmentation of phytobezoar may be considered as long as the patient has a normal gastric emptying function and no distal obstruction. The surgical options are either laparoscopic or open via fragmentation and milking the bezoar. The management was even more tricky in our situation as the patient had presented during the peak of COVID-19 pandemic. There was observed high morbidity and mortality with patients suspected or confirmed with COVID-19. The giant HH had compromised the patients lung capacity. Hence, we wanted to try conservative management to overcome this difficult time. A multidisciplinary approach with surgeons, gastroenterologist, and dietitians helped us successfully managing this patient conservatively.

## Conclusion

Phytobezoars in a giant hiatus hernia can be successfully treated without surgery in some selected cases with a multidisciplinary team approach. We wanted to raise awareness of this rare pathology.
